# Association of workload and practice of respectful maternity care among the healthcare providers, before and during the early phase of COVID-19 pandemic in South Western Nepal: a cross-sectional study

**DOI:** 10.1186/s12913-023-09561-x

**Published:** 2023-05-24

**Authors:** Alpha Pokharel, Junko Kiriya, Akira Shibanuma, Ram Chandra Silwal, Masamine Jimba

**Affiliations:** 1 Green Tara Nepal, Kathmandu, Nepal; 2 Department of Community and Global Health, Graduate School of Medicine, The University of Tokyo, Tokyo, Japan

**Keywords:** Respectful maternity care, Disrespect and abuse, Workload, Client provider ratio, Nepal, COVID-19

## Abstract

**Introduction:**

Respectful maternity care is an approach that involves respecting women’s belief, choices, emotions, and dignity during the childbirth process. As the workload among maternity care workforce affects intrapartum quality care, respectful maternity care might have also been affected, particularly during the pandemic. Thus, this study was conducted to examine the association between workload among healthcare providers and their practice of respectful maternity care, before and during the early phase of pandemic.

**Methods:**

A cross-sectional study was conducted in South Western Nepal. A total of 267 healthcare providers from 78 birthing centers were included. Data collection was done through telephone interviews. The exposure variable was workload among the healthcare providers, and the outcome variable was respectful maternity care practice before and during the COVID-19 pandemic. Multilevel mixed-effect linear regression was used to examine the association.

**Results:**

The median client-provider ratio before and during the pandemic was 21.7 and 13.0, respectively. The mean score of respectful maternity care practice was 44.5 (SD 3.8) before the pandemic, which was decreased to 43.6 (SD 4.5) during the pandemic. Client-provider ratio was negatively associated with respectful maternity care practice for both times; before (Coef. -5.16; 95% CI -8.41 to -1.91) and during (Coef. -7.47; 95% CI -12.72 to -2.23) the pandemic.

**Conclusions:**

While a higher client-provider was associated with a lower respectful maternity care practice score both before and during the COVID-19 pandemic, the coefficient was larger during the pandemic. Therefore, workload among the healthcare providers should be considered before the implementation of respectful maternity care, and more attention should be given during the pandemic.

**Supplementary Information:**

The online version contains supplementary material available at 10.1186/s12913-023-09561-x.

## Introduction

Respectful maternity care is an approach based on the principle of ethics and the fundamental rights of women. Respectful maternity care includes respecting women’s beliefs, independence, choices, emotions, and dignity while providing maternity care [1]. Implementing respectful maternity care in birthing centers reduces unnecessary medical interventions such as episiotomy and fundal pressure. Respectful maternity care also improves women’s satisfaction with care and decreases disrespect and abuse [2]. Whereas, disrespectful treatment or negligence during childbirth endangers both mothers’ and newborns’ health and decreases women’s future use of health facilities [2]. A large number of women experience disrespect and abuse during childbirth, with the global prevalence varying between 15% and 98% [3]. Some common forms of disrespectful maternity care prevalent worldwide are physical abuse, verbal abuse, refusal to provide pain relief, abandonment, poor communication, and lack of privacy [4].

As a response to personal safety and security during the COVID-19 pandemic, some of the respectful maternity care practices deteriorated more than before. The rapidly changing guidelines and enhanced infection prevention measures hindered healthcare providers from implementing respectful maternity care [5]. For instance, restriction of labor companion and breastfeeding, reduced emotional and physical support for women, compromised standards of care, and increased medically unjustified cesarean section during the COVID-19 pandemic [5–7].

An adequate supply of health human resources is essential to delivering respectful maternity care [8]. In a qualitative study, healthcare providers reported insufficient staff members, high patient loads, and inadequate facilities as barriers for better maternity care practice [9]. Workload among healthcare providers has caused insufficient and unnecessary patient care [2, 10]. A higher workload among healthcare providers at the birthing centers has decreased interpersonal communication with the women [11]. Increased institutional delivery rates but the ongoing shortage of healthcare professionals has placed a greater burden on healthcare providers [12]. The inclusion of tasks other than the clinical aspect, such as administrative, monthly reporting, and field works, has further increased their workload [13]. Consequently, the tasks that do not directly affect the health of women and newborns are often ignored [2, 10]. The increased workload has also called upon many malpractices in Low-and Middle-income Countries(LMICs), such as unnecessary episiotomy and fundal pressure [11]. Moreover, the increased workload among healthcare providers has negatively affected the quality of care they provide [14].

Nepal has made substantial progress in improving maternal health care access and utilization [15]. Despite this, progress is still required to reach universal access to sexual and reproductive health services by 2030. Along with socio-demographic factors, the low quality of care is also a significant barrier to maternal healthcare access and utilization in Nepal [16, 17]. As Nepalese women bypass the primary healthcare centers to have child delivery at the tertiary centers, these centers are overburdened with clients [18, 19]. These tertiary health centers were more burdened with clients during the COVID-19 pandemic [20]. Healthcare providers in Nepal were found to have a discriminatory attitude towards COVID-19 patients and feared of acquiring the COVID-19 infection from them. The fear of acquiring COVID-19 infection was associated with low job satisfaction [21]. These situations definitely affected the standard of practice among the healthcare providers. Also, the mandatory infection prevention measures introduced during the COVID-19 pandemic further increased the workload among healthcare providers. The increment in workload during the pandemic significantly affected maternal healthcare quality [20].

Respectful maternity care should be understood from both providers’ and mothers’ sides as it is crucial for its effective implementation [22]. However, most studies have focused on the mothers’ side, and research on the providers’ side is still lacking [3, 9]. Since healthcare providers’ scarcity was negatively associated with the quality of skilled birth care [23], knowing if the workload among healthcare providers affects the respectful maternity care practice becomes crucial. It is also important to assess the influence of the COVID-19 pandemic on respectful maternity care practice, since the COVID-19 pandemic has further burned the workload among healthcare providers and negatively affected maternity care [20]. Thus, this study was conducted to examine the association of workload among healthcare providers and their respectful maternity care practice, both before and during the COVID-19 pandemic.

## Methods

### Study design and settings

A cross-sectional study was conducted in South Western Nepal. The study area included four districts: Rupandehi, Kapilvastu, Nawalparasi (West of Bardaghat Susta), and Nawalparasi (East of Bardaghat Susta). Nawalparasi (East of Bardaghat Susta) is located in Gandaki Province of Nepal, whereas the other three districts are located in Lumbini province [24]. The study area includes 16 urban municipalities, 21 rural municipalities, and 1 sub-metropolitan city. The study area covers 4,776 square kilometers and has a population of 2,084,054 [25]. Among the total government-run health facilities in the study site, 79 had birthing centers, and all of them were included in this study.

In Nepal, birthing services are available at Health Post (HP), Primary Health care Center (PHCC), and Hospital. At HP, basic obstetric care services are available, which includes management of no-risk labor, providing obstetric first aid, making an appropriate referral, and arranging transport. At the PHCC level, BEmONC (basic emergency obstetric and newborn care) is available. It provides prevention and treatment of hemorrhage, sepsis, eclampsia, and infection, and the management of prolonged labour along with basic obstetric care. At the hospital level, Comprehensive Emergency Obstetric and Newborn Care (CEmONC) is available, which includes management BEmONC along with cesarean sections, anesthesia, and blood transfusion [26].

These districts were purposively selected as a study setting because of their unsatisfactory maternal health status [27, 28], and the impact of COVID-19 [29]. The burden of the COVID-19 pandemic in these districts was among the highest in Nepal [29, 30]. The rate of institutional delivery was found to be low in Kapilvastu, Nawalparasi (West of Bardaghat Susta), and Nawalparasi (East of Bardaghat Susta) [27, 28]. Rupandehi was selected because of its high volume of institutional delivery, obstetric complications [27].

### Participants

In Nepal, at the HP and PHCC levels, an Auxiliary Nurse Midwife (ANM) and Staff Nurse are posted in the birthing Center. In the hospital level birthing center, one doctor (MBBS, OB/GYN diploma) works with staff nurses and ANM. For this study, the definition of a healthcare provider is a healthcare professional who provides continuous maternity care to women. In the context of Nepal, doctors usually only offer management of high-risk labor cases and management of labor complications.; therefore, doctors were not included in this study.

Healthcare providers were eligible to participate in the study if they had more than two years of total birthing center experience and worked at the present birthing center for more than three months. The duration of work experience was considered to ensure familiarization with the context and work culture [31]. Healthcare providers were eligible to participate in the study regardless of age and education level.

### Variables

#### Exposure variable

The exposure variable was the workload among the healthcare providers. Both subjective and objective measures of workload were assessed. The subjective workload was assessed at individual provider level with the National Aeronautics and Space Administration Task Load Index (NASA TXL) scale [24]. It is a scale with a six-item questionnaire that measures the level of mental demand, physical demand, temporal demand, performance, effort, and frustration for the current work [32, 33]. It consists of a two-part evaluation process: weights and ratings, and its total score range from 0 to 100. A higher score represents a higher workload [33, 34].

The objective workload was assessed by the client-provider ratio [35], and the total number of deliveries at the health facility level [11]. For the number of deliveries, the healthcare providers were asked to recall the approximate total number of deliveries attained in previous month of data collection. The client-provider ratio was assessed at health facility level and was calculated by dividing the total number of births in the health facility by the total number of healthcare providers during that period.

In Nepal, restrictive measures taken against COVID-19 influenced the client-provider ratio, as the number of institutional births decreased in Nepal [19]. To avoid the influence, the client-provider ratio was calculated in two time periods: six months before and after the COVID-19 pandemic declaration by WHO on March 11, 2020 [36]. Converting the Nepalese calendar to the Gregorian calendar, the two periods for calculating the client-provider ratio were: July/August 2019 to January/February 2020 (before COVID-19 pandemic declaration) and February/March 2020 to June/July 2020 (after COVID-19 pandemic declaration) [37]. To better interpret the client-provider ratio in the regression analysis, the client-provider ratio of six months was converted to per day.

#### Outcome variable

The outcome variable in this study was healthcare providers’ practice of respectful maternity care. It was assessed using a questionnaire adapted from the performance standard for respected maternity care prepared by the Maternal and Child Health Integrated Program [38]. This tool was chosen for this study because it was used in other similar LMICs [39, 40]. Also, it was adapted from Bowser and Hills’s landscape analysis on disrespect and abuse in facility-based birth, which was developed using evidence from multiple countries [8]. The questionnaire is comprised of 7 domain and 27 items: non-abusive care (6 items), consented care (9 items), confidential care (3 items), dignified care (3 items), non-discriminative care (2 items), non-abandonment care (3 items), and non-detention care (1 item) [38]. As all the included birthing centers provided free maternity service to the clients, the non-detention domain (detention in a health facility due to inability to pay hospital bills) was removed from the questionnaire [41]. Each item has three responses: always (2 points), sometimes (1 point), and never (0 point). The scores of all items were summed to compute the total score. The possible score ranges from 0 to 52, and a higher score represents the better practice of respectful maternity care.

The maternity care practice was also affected by the COVID-19 in Nepal [20]. To incorporate the issue, the healthcare providers were asked to report the practice of each item of respectful maternity care before (July/August 2019 to January/February 2020) and during the early phase of COVID-19 pandemic (February/March 2020 to June/July 2020) [37].

### Confounders and covariates

Potential confounders and covariates were added to the study. Healthcare providers’ job positions [8, 42] and education level [11, 43] were included as confounders. Awareness of respectful maternity care, SBA training, age, being tested positive for COVID-19, and years of job experience were included as covariates [11]. Healthcare providers were considered to be aware of respectful maternity care if they had ever heard or read about it in the past.

### Validity and reliability of the scale

Permission was obtained from the concerned organization for the use of the questionnaires. As the questionnaires were not available in the Nepali language, they were checked for cultural adaptation through translation and back translation to the Nepali language and pre-testing of scale among the healthcare providers [44]. Two native speakers of the Nepali language did the translation, one of whom was familiar with the subject matter, and the other was not. The translated documents were reviewed and verified by a subject matter specialist. Two other native Nepali speakers did back translations, and the final version was reviewed and verified by a subject matter specialist [45]. The respectful maternity care practice questionnaire had not been tested for validity and reliability among healthcare providers, and was not used in Nepal before. Thus, content validity was ensured through three experts’ opinions, two online Focus Group Discussions (FGDs) among the healthcare providers, and the pre-test of the questionnaires among the healthcare providers. Two Skilled Birth Attendant (SBA) trainers from the National Health Training Center and 1 Associate Professor of the maternal and child health department from B.P Koirala Institute of Health Science were contacted for their expert opinion. The online FGDs were conducted among 10 healthcare providers from Ranjani health post and Rangeli hospital, Morang (5 healthcare providers each). One moderator facilitated the interview, and one note-taker was present during the FGDs. The FGDs were conducted to assess the content validity of the questionnaire in context to Nepal. The pre-test was conducted among 40 healthcare providers from the Nobel Medical College and Teaching Hospital birthing center and Katari Hospital. After expert opinion, FGDs, and pre-test of the questionnaire, modifications were made to the language structures. Also, 1 item on non-detention care from the total 27 items was removed based the expert’s judgment and FGDs among the healthcare workers. Since all the birthing centers included in this study had a safe-motherhood program where the clients do not have to pay for any service related to labor and childbirth, the non-detention standard (women’s not being detained in hospital if they were not able to pay the hospital bills) was removed from the questionnaire [41]. The healthcare providers for FGD and pre-test were not from the study setting, and were not included in the study. The data from the pre-test was not included in the final data analysis. Cronbach’s alpha was calculated to measure the internal consistency of the respectful maternity care practice questionnaire [44]. The Cronbach’s alpha coefficient was above 0.80 for each of the six domains, and 0.93 for the whole questionnaire (26 item).

### Data collection

Data were collected from September to October 2020. Data were collected through telephone using interviewer-administered questionnaire and response was simultaneously entered into Google forms. The healthcare providers were contacted beforehand for verbal consent and data collection schedule. The research assistants were hired, and trained to obtain verbal consent and to conduct telephone-based interviews. It took approximately 25 min to explain the study and interview the healthcare providers. The data for the client-provider ratio were obtained from the records of the health authority.

### Data analysis

Descriptive statistics was performed to present the background characteristics of health facilities and healthcare providers, and practice of respectful maternity care among the healthcare providers. A paired t-test was performed to compare the practice score of respectful maternity care before and during the COVID-19 pandemic. A two-level, mixed-effect linear regression analysis was performed with a random intercept at the health facility level. Two null and full models were used for the sub-category of the outcome variable: respectful maternity care practice before and during the COVID-19 pandemic. The intraclass correlation coefficient was used to compare the proportion of variance caused by the random intercept at the health facility level. The significance level was set at 0.05. Google sheet was used for data organization and filtering. Data were exported to R Studio version 1.2.5001 for statistical analyses.

### Research ethics

An ethics approval and procedure to obtain informed verbal consent was obtained from the Research Ethics Committee, Graduate School of Medicine, the University of Tokyo, Japan (serial number: 2020101NI), and Nepal Health Research Council, Nepal (ERB protocol registration number: 524/2020 MT). Permission for data collection was obtained from District Health Offices, district hospitals, and municipality hospitals. Permission to use healthcare providers’ phone numbers was obtained from them through the head of health facilities. Participation in this study was voluntary. Confidentiality was assured, and informed verbal consent was taken from each healthcare provider. Documentation of the date and time of consent was done. The audio recording of the telephone interview and the consent process were not done. All methods were carried out in accordance with relevant guidelines and regulations.

## Results

### Characteristics of health facility

Out of 79 birthing centers, 78 were included in the study. One province hospital was excluded from the study due to administrative issues. Kapilvastu district had the highest number of health facilities (n = 28), and the Nawalparasi (West of Bardaghat Susta) district had the least (n = 13). About three-quarters of the health facilities were health posts (Table 1).

**Table 1:** Characteristics of health facilities (n = 78)

Level of Health Facility	Districts				Total
	Kapilvastu	Rupandehi	Nawalparasi (East of Bardaghat Susta)	Nawalparasi (West of Bardaghat Susta)	
Health Post	23	16	10	8	57
PHCC^a^	2	5	4	2	13
Municipality Hospital	2	0	0	2	4
District Hospital	1	1	1	1	4
Total	28	22	15	13	78

^a^PHCC: Primary Health Care Center.

### Background characteristics of the healthcare providers

A total of 318 healthcare providers were working at the 78 birthing centers. Among them, 51 healthcare providers were unable to reach through phone. As a result, 267 healthcare providers completed the interview and were included in data analyses. The least number of healthcare provider recruited from a health facility was one, whereas the highest was 11. There were no missing data. Table 2 presents the summary of their background characteristics. The majority of the healthcare providers had undergone Auxiliary Nurse Midwife (ANM) education (70.4%), and SBA training (73.4%), worked as an ANM (88.4%), and worked at a health post (63.3%). About 80% of the healthcare providers had never heard of or read about respectful maternity care in the past, and around 7.0% of them had been tested positive for COVID-19 (Table 2).

**Table 2:** Background characteristics of the healthcare providers (n = 267)

Characteristics	n (%)	Mean (SD)
**Age (years)**		30.9 (7.7)
**Education**		
Auxiliary Nurse Midwife	188 (70.4)	
Proficiency Certificate Level Nursing	64 (24.0)	
Bachelor of Nursing	15 (5.6)	
**Occupation**		
Auxiliary Nurse Midwife	236 (88.4)	
Staff Nurse	30 (11.2)	
Nursing Officer	1 (0.4)	
**Total work experience (years)**		8.3 (6.8)
**Experience at the present birthing center (years)**		3.5 (4.2)
**SBA training**		
Yes	196 (73.4)	
No	71 (26.6)	
**Ever heard/read about respectful maternity care**		
Yes	48 (18.0)	
No	219 (82.0)	
**COVID-19 test (PCR test) in past**		
Positive	18 (6.7)	
Negative	249 (93.3)	
**District**		
Kapilvastu	83 (31.1)	
Rupandehi	71 (26.6)	
Nawalpur	56 (21.0)	
Parasi	57 (21.3)	
**Level of health facility**		
Health Post	169 (63.3)	
Primary Health Care Center	44 (16.5)	
Municipality Hospital	20 (7.5)	
District Hospital	34 (12.7)	

### Workload among the healthcare providers

The healthcare providers’ mean total subjective workload score was 77.7 (range: 30–100). The median client-provider ratio of six months before and during the COVID-19 pandemic was 21.7 (IQR 9.7 to 52.2) and 13.0 (IQR 6.2 to 28.4). The mean number of deliveries attended by a healthcare provider in the last month was 13.6 (SD 19.6) (Table 3).

**Table 3:** Client-provider ration and workload among healthcare providers

Characteristics	Median (1Q-3Q)	Range
Client-provider ratio of 6 months, before the COVID-19 pandemic (n = 263)	21.7 (9.7–52.2)	0.3–189.0
Client-provider ratio of 6 months, during the COVID-19 pandemic (n = 263)	13.0 (6.2–28.4)	1.2–144.0
Number of deliveries attended in last month (n = 267)	9.0 (4.0–15.0)	0.0-150.0
A score of subjective workload [NASA TXL Scale] (n = 267)	78.3 (70.0-87.8)	30–100

### Respectful maternity care practice

During the COVID-19 pandemic, mean total score of respectful maternity care decreased to 43.6 (SD 4.5) from 44.5 (SD 3.8) (See Supplementary Fig. 1). Table 4 presents the score of practices under each domain of respectful maternity care. For the abuse-free care domain, all healthcare providers never restrained the women during labor. However, 27.3% of them sometimes had to physically or verbally abuse the women. Also, 59% of them always provided pain and comfort relief to the women in labor. The practice of always touching the women in a culturally appropriate way was 95.1% before the COVID-19 pandemic and 89.5% during the pandemic. All healthcare providers never separated the baby with the mother, both before and during the pandemic.

**Table 4:** Item score of respectful maternity care practice (n = 267)

Questions	Before COVID-19 pandemic			During COVID-19pandemic			*p*-value
	Always (%)	Sometimes (%)	Never (%)	Always (%)	Sometimes (%)	Never (%)	
**Abuse-free care**							
Never physically or verbally abused	72.2	27.3	0.0	72.0	28.0	0.0	0.318
Never restrained physically	100.0	0.0	0.0	100.0	0.0	0.0	0.318
Touched in a culturally appropriate way	95.1	4.9	0.0	89.5	10.5	0.0	< 0.001
Never separated the baby from mother^a^	98.5	1.5	0.0	96.2	3.8	0.0	0.057
Never denied food and fluids^a^	97.0	3.0	0.0	97.0	3.0	0.0	1.000
Provided comfort/pain relief	58.8	23.9	17.2	59.1	17.0	23.9	0.318
**Right to information, informed consent, and choice**							
Introduced self to women and her companion	34.8	54.6	10.4	32.5	52.3	15.2	< 0.001
Encouraged companion to stay with women	62.5	24.7	12.8	49.0	23.5	27.5	< 0.001
Encouraged to ask questions	91.0	9.0	0.0	85.0	14.2	0.8	< 0.001
Responded questions promptly and politely	92.8	7.2	0.0	86.1	13.9	0.0	< 0.001
Explained the procedure	90.6	9.0	0.4	84.0	14.2	1.8	< 0.001
Provided periodic update	93.2	6.8	0.0	85.7	12.7	1.6	< 0.001
Allowed to move during labor	53.5	42.6	3.7	54.0	42.3	3.7	0.318
Allowed to assume the birth position of choice	19.1	32.9	48.0	19.4	32.2	48.4	0.990
Obtained consent before the procedure	91.7	7.4	0.7	91.3	8.0	0.7	0.318
**Confidential care**							
Stored file in locked cabinets	19.1	47.9	33.0	19.0	47.7	33.3	0.157
Used curtain or visual barrier	56.6	34.8	8.6	56.2	38.0	7.8	0.655
Used drapes and covering	70.7	26.5	2.6	71.1	26.3	2.6	0.564
**Dignified care**							
Spoke politely to women	94.0	5.6	0.4	93.0	6.6	0.4	0.083
Allowed non-harmful cultural practice	95.8	4.2	0.0	95.5	4.5	0.0	0.318
Never insulted and intimidated	94.3	5.7	0.0	94.3	5.7	0.0	1.000
**Equitable care**							
Spoke in an understandable language	92.5	7.5	0.0	92.5	7.5	0.0	1.000
Never disrespected & discriminated the women	98.8	1.2	0.0	98.5	1.5	0.0	0.318
**Non-abandon care**							
Encouraged to call if required	96.4	3.3	0.3	92.5	7.2	0.3	0.001
Came quickly when called	67.7	32.3	0.0	64.8	35.2	0.0	0.004
Never left women alone	56.7	41.8	0.5	54.0	42.3	3.7	0.001

^a^ unless medically indicated

Regarding the right to information and informed choice care domain, the practice of always allowing a labor companion was 62.5% before the pandemic, and 49.0% during the pandemic. Statistically significant differences in practices for two time points were noticed for: always allowing labor companion, always introducing self to the women and her companion (before and during the pandemic: 34.8% and 32.5%), always encouraging women to ask questions (before and during the pandemic: 91.0% and 85.0%), respond the questions promptly (before and during the pandemic: 92.8% and 86.1%), explain the producers (before and during the pandemic: 90.6% and 84.0%), and provide periodic updates during the COVID-19 pandemic (before and during the pandemic: 93.2% and 85.7%). Allowing women to choose the birth position was never practiced by approximately half of the healthcare providers, both before and during the pandemic.

For the confidential care domain, about one-third of the healthcare providers never stored women’s files in the locked cabinet, both before and during the pandemic. Around half of the healthcare providers sometimes or never used curtains for procedures, both before and during the pandemic.

For non-abandon care domain, 96.4% of the healthcare providers always encouraged the women to call before the COVID-19 pandemic, but it was 92.5% during the pandemic. When called, 67.7% of the healthcare providers always came quickly before the pandemic, but 64.8% came during the pandemic. While the practice of never always leaving the women alone during labor was 56.7% before the pandemic, it was 54.0% during the pandemic.

### Factors associated with respectful maternity care practice

Table 5 presents the results of the multilevel mixed-effect linear regression analysis with a random intercept at the health facility level, for factors associated with respectful maternity care practice by the healthcare providers. The results are presented in two models: respectful maternity care practice before the COVID-19 pandemic and during the pandemic. There are also two null models accompanying each of these full models. Education and total years of experience were excluded from the final model, as they were found to be collinear with occupation and total years of experience, respectively [46].

**Table 5:** Multilevel mixed-effects linear regression analysis for respectful maternity care practice among the healthcare providers

Predictors	Respectful Maternity Care Practice					
	**Before the COVID-19 pandemic**		**P-value**	**During the COVID-19 pandemic**		**p-value**
	**Null model**	**Final Model (n = 267)**		**Null model**	**Final Model (n = 267)**	
		**Estimates (CI)**			**Estimates (CI)**	
**Healthcare provider characteristics (Fixed effect)**						
**The subjective measure of workload**		0.02 (-0.02 to 0.05)	0.376		0.02 (-0.02 to 0.06)	0.246
**Experience at the present birthing center**		-0.04 (-0.15 to 0.07)	0.450		-0.02 (-0.14 to 0.11)	0.784
**Occupation**						
Auxiliary Nurse Midwife		Reference			Reference	
Staff Nurse		-0.12 (-1.62 to 1.39)	0.879		-0.004 (-1.68 to 1.69)	0.996
**Age**		0.08 (0.02 to 0.14)	0.011		0.07 (0.002 to 0.14)	0.037
**Ever heard/read about RMC**						
No		Reference			Reference	
Yes		0.68 (-0.37 to 1.73)	0.203		1.38 (0.17 to 2.59)	0.055
**Skilled Birth Attendant training**						
No		Reference			Reference	
Yes		-0.20 (-1.15 to 0.75)	0.684		-0.36 (-1.43 to 0.71)	0.513
**COVID-19 test (PCR test) in past**						
Negative/ Not tested		-			Reference	
Positive		-			-3.18 (-5.06 to -1.30)	0.001
**Number of deliveries attended last month**		-0.02 (-0.04 to 0.01)	0.129		-0.02 (-0.04 to 0.01)	0.236
**Health facility level characteristics (n = 78)**						
**Client-provider ratio per day**						
Before the COVID-19 pandemic*		-5.16 (-8.41 to -1.91)	0.002			
During the COVID-19 pandemic					-7.47 (-12.72 to -2.23)	0.005
**Level of health facility**						
Health Post		0.48 (-1.39 to 2.36)	0.614		-0.12 (-2.41 to 2.18)	0.921
Primary Health Care Center		-0.55 (-2.69 to 1.58)	0.611		-1.93 (-4.57 to 0.71)	0.151
Hospital (Municipality + District)		Reference			Reference	
**Random effect**						
**Health facility level variance (SD)**	5.04 (2.24)	3.25 (1.80)		8.36 (2.89)	5.60 (2.36)	
**ICC (%)**	34.0	26.0		40.0	33.0	

CI: Confidence Interval, SD: Standard Deviation, ICC: Intraclass Correlation Coefficient, Before the COVID-19 pandemic: July/August 2019 to January/February 2020, During the COVID-19 pandemic: February/March 2020 to June/July 2020

The healthcare providers’ characteristics were considered as level 1 variable, and health facility characteristics were considered as level 2 variable. The health facilities in the null model explained 34.0% variance for the respectful maternity care practice before the COVID-19 pandemic. After controlling for exposure variables, 26% of the variation was explained by the health facilities. The null model explained 40% variance of practice among the health facilities, during the COVID-19 pandemic. After controlling the exposure variables, it was 33.0%.

Age of the healthcare providers was positively associated with respectful maternity care practice before (Coef. 0.08; 95% CI 0.02 to 0.14) and during (Coef. 0.07; 95% CI -0.002 to 0.14) the COVID-19 pandemic. Being tested positive for COVID-19 in the past was negatively associated with respectful maternity care practice during the COVID-19 pandemic (Coef. -3.18; 95% CI -5.06 to -1.30). The client-provider ratio per day was negatively associated with respectful maternity care practice before (Coef. -5.16; 95% CI -8.41 to -1.91) and during (Coef. -7.47; 95% CI -12.72 to -2.23) the COVID-19 pandemic.

## Discussions

### Principal findings

This study investigated the association between workload and the practice of respectful maternity care among healthcare providers in South Western Nepal, before and during the early phase of COVID-19 pandemic. While the workload among the healthcare providers was negatively associated with respectful maternity care practice, both before and during the pandemic, the effect of workload on the practice of respectful maternity care was larger during the pandemic.

The coefficient between workload and practice of respectful maternity care was larger during the pandemic (Coef. -7.47; 95% CI -12.72 to -2.22) than before (Coef. -5.16; 95% CI -8.41 to -1.91). In a high workload setting, healthcare providers often ignore the procedures that do not immediately impact the mother and baby’s life [47, 48]. These high workload settings are often located in urban areas, where COVID-19 cases are high [5, 36]. Since healthcare providers want to prevent themselves from acquiring COVID-19 infection, they avoid respectful maternity care practices such as client communication and labor companionship [5]. A qualitative survey done among healthcare providers of multiple nation also found that the restrictive measure taken during the pandemic could negatively affect the respectful maternity care [7]. As a result, the effect of workload on respectful maternity care practice might have been greater during the pandemic in this study. A study from Nepal found that the fear of COVID-19 among healthcare providers resulted in low work satisfaction, low burnout, and low fatigue [21]. Another study from Nepal also found a decrease in labor companionship during the pandemic [20]. Therefore, decrement of workload among healthcare providers should be considered more during the pandemic.

The variance among each health facility for respectful maternity care practice increased more during the pandemic. The difference in client-provider ratio found among the health facilities from this study, could be a possible reason for the variance of respectful maternity care across the health facilities. It was also found from the national data that the decline in delivery cases varied across the type of health facility in Nepal [37]. While 50% or more of local health facilities were closed during the early lockdown period for child delivery, almost all tertiary healthcare centers were open [37]. Strengthening the quality of local health facilities can increase child delivery cases there, which will help distribute the client-provider ratio among health facilities [49]. As a result, respectful maternity care can be practiced, even during the pandemic.

Increased age among the healthcare providers was positively associated with the respectful maternity care practice. A possible explanation could be, with age healthcare providers become more experienced with maternity care. They become more skillful in satisfying women’s need, and respecting her desires [11].

Healthcare providers who tested positive for COVID-19 had lower respectful maternity care practice scores. The practice of respectful maternity care has been lower in most parts of the world [3, 50]. A qualitative study found that the COVID-19 pandemic further decreased the practice of respectful maternity care [7]. This might be due to stigma placed by the community for being the source of infection [51, 52]. A study done among healthcare providers working in COVID-19 hospitals of Nepal found that the healthcare providers were facing stigma for being the source of infection, and the stigma was associated with anxiety among the healthcare providers [52]. To avoid being stigmatized, they might have avoided client interaction and communication, which are components of respectful maternity care practices such as [52, 53]. Also, other possible reasons could be decreased mental and physical performance due to stigma, psychological distress, and diseases [54, 55]. Therefore, more physical and mental health support to healthcare providers should be provided to improve their performance [56].

### Strengths and weaknesses

This study has several strengths. It is one of the first study on healthcare providers’ workload and its association with respectful maternity care during the COVID-19 pandemic. As this study included all the health facilities in the study area, it provides an overview of all levels of health facilities and all the cadre of healthcare providers in Nepal.

It also has several limitations. The outcome might have been affected by social desirability bias. In order to overcome it, assurance of anonymity and confidentiality was provided. Also, the healthcare providers were well explained about the objectives of the study and its possible impact on the scientific literatures. The data related to respectful maternity care practice before the pandemic depended on the healthcare provider’s memory, which might have introduced a measurement error in the outcome assessment. Since the COVID-19 pandemic occurred during the data collection, the researcher had to rely on the self-reporting data for before the pandemic period. There is also possibility of recall bias. To help healthcare provider remember the event well, they were initially asked about their current practice (during the early phase of pandemic) and then asked if there were any changes before February/March 2020 (before the pandemic). They were also given adequate time to think [57]. The visual rating of the NASA TXL scale was changed to auditory description, as the data collection was done through telephone for the prevention of spread of COVID-19 infection. The change of scale from visual to auditory could have caused over-reporting or under-reporting of the workload. This could be the reason why it did not show strong evidence for the association with the respectful maternity care practice [58]. The content validity of the practice standard was ensured through the judgement from expert and healthcare workers, there was no quantitative calculations such as factor analysis and Content Validity Ratio (CVR) done [59, 60] .

## Conclusions

While a higher client-provider ratio was associated with a lower respectful maternity care practice score both before and during the COVID-19 pandemic, the coefficient was larger during the pandemic. Also, the variance among the health facilities for respectful maternity care was increased more during the COVID-19 pandemic. The findings of this study call for the decrement in the client-provider ratio for better respectful maternity care practice, especially during the pandemic. However, supplying health human resources per the population demand, especially during the pandemic, may be difficult in a resource-limited setting. The number of deliveries at local health facilities can be increased by improving the quality of care, particularly during the pandemic. This could help equal distribution of client-provider and practice respectful maternity care, even during the pandemic. Further, the healthcare providers who were tested positive for COVID-19 had lower respectful maternity care practice scores. Therefore, additional physical and mental health support to the healthcare providers should be considered to improve their respectful maternity care practice, particularly during the pandemic.

## Electronic supplementary material

Below is the link to the electronic supplementary material.

Supplementary Material 1

Supplementary Material 2

## Data Availability

The datasets generated and/or analysed during the current study are not publicly available due to confidentiality of the participant data, but data are available from the corresponding author on reasonable request.
