# Supplementary material for: Association of workload and practice of respectful maternity care among the healthcare providers, before and during the early phase of COVID-19 pandemic in South Western Nepal: a cross-sectional study
Source: BMC Health Serv Res. 2023 May 24;23:538. doi: 10.1186/s12913-023-09561-x (PMC10208183; doi:10.1186/s12913-023-09561-x)
Supplement: Supplementary file 1 — Supplementary Material 1 [file 12913_2023_9561_MOESM1_ESM.docx]

**Supplementary figure 1. Score of respectful maternity care practice and its domains (n=267)**


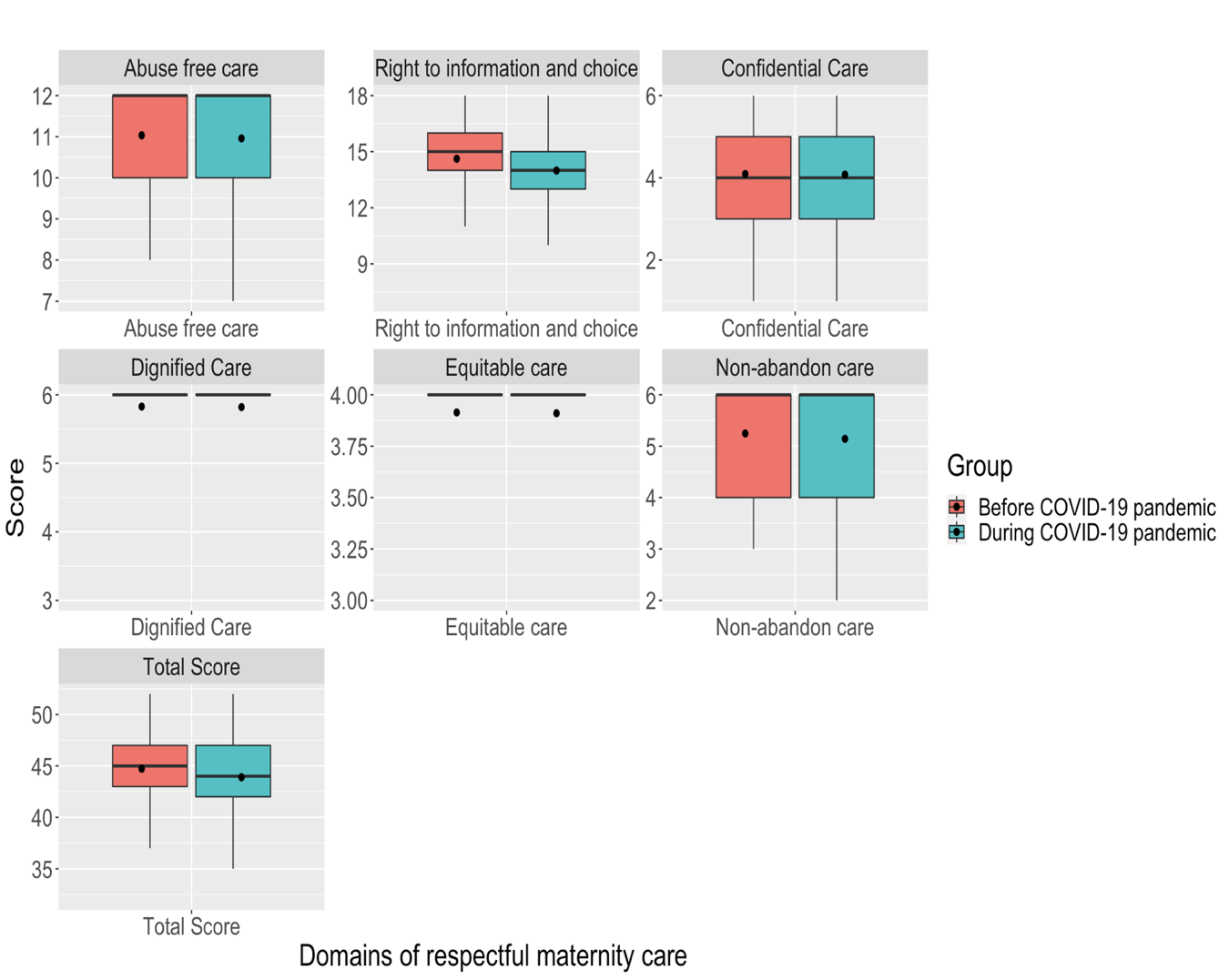


Before the COVID-19 pandemic: July/August, 2019 to January/February, 2020

During the COVID-19 pandemic: February/March, 2020 to June/July, 2020
