# Supplementary material for: Association of workload and practice of respectful maternity care among the healthcare providers, before and during the early phase of COVID-19 pandemic in South Western Nepal: a cross-sectional study
Source: BMC Health Serv Res. 2023 May 24;23:538. doi: 10.1186/s12913-023-09561-x (PMC10208183; doi:10.1186/s12913-023-09561-x)
Supplement: Supplementary file 2 — Supplementary Material 2 [file 12913_2023_9561_MOESM2_ESM.pdf]

Association between workload and practice of respectful maternity care among the healthcare providers in the south-western part of Nepal: a cross-sectional study

Survey Questionnaire

* Required

Survey Questionnaire in english

1. Identification of interviewee (phone number) *

2. Name of district: *

3. Name of Health Facility *

4. Township *

   Mark only one oval.

   - Sub-metropolitan City
   - Municipality
   - Rural municipality

5. Level of health facility *

Mark only one oval.

- ( ) Health Post
- ( ) Primary Health Care Center
- ( ) District Hospital
- ( ) Province Hospital
- ( ) Urban Health Clinic
- ( ) Community Hospital
- ( ) Independent Birthing Center
- ( ) others

Demographic, occupation and work-related information

6. How old are you? (completed years) *

____

7. What is your highest completed education level? (profession related) *

Mark only one oval.

- ( ) Maternal and child health work training
- ( ) Auxillary Nurse Midwife training
- ( ) Proficiency Certificate level Nursing
- ( ) Bachelor of Nursing
- ( ) Postgraduate Nursing
- ( ) Other paramedical training

8. What is your current job position? *

Mark only one oval.

- ( ) Maternal and child health worker
- ( ) Auxiliary Nurse Midwife
- ( ) Staff Nurse
- ( ) Nursing Officer
- ( ) Nursing Administrator
- ( ) Others

9. How many years of work experience do you have? *

____

10. How many years of work experience do you have at the current birthing center? *

____

11. Have you taken the Skilled Birth Attendant training? *

Mark only one oval.

- ( ) Yes
- ( ) No

12. Have to ever heard, trained or read about Respectful maternity care/ disrespect and abuse during maternity care? *

Mark only one oval.

- ( ) Yes
- ( ) No
- ( ) Maybe

13. If yes, where did you hear or read about it?

Mark only one oval.

- ⬭ at college/school/university
- ⬭ at training
- ⬭ others

14. Have you been tested positive for COVID-19? If you want to answer *

Mark only one oval.

- ⬭ Yes
- ⬭ No
- ⬭ don't want to answer/reveal

Healthcare provider's work-related data

15. Number of deliveries you attended in last 1 week *

____

16. Number of deliveries you attended in last 1 month

____

17. Number of deliveries you attended in last 6 month

____

18. Number of extra shifts you did in last 1 week *

____

19. Number of extra shifts you did in last 1 month

Mark only one oval.

- Option 1

20. Number of extra shifts you did in last 6 month

Mark only one oval.

- Option 1

Assessment of perceived workload

21. Which of the two item is more important contributor to your workload *

Mark only one oval.

- Effort
- Performance

22. Which of the two item is more important contributor to your workload *

Mark only one oval.

- Temporal demand
- Frustation

23. Which of the two item is more important contributor to your workload *

Mark only one oval.

- Frustation
- Effort

24. Which of the two item is more important contributor to your workload *

Mark only one oval.

- Temporal demand
- Effort

25. Which of the two item is more important contributor to your workload *

Mark only one oval.

- Physical demand
- Frustation

26. Which of the two item is more important contributor to your workload *

Mark only one oval.

- Performance
- Mental demand

27. Which of the two item is more important contributor to your workload *

Mark only one oval.

- Performance
- Frustation

28. Which of the two item is more important contributor to your workload *

Mark only one oval.

- Physical demand
- Temporal demand

29. Which of the two item is more important contributor to your workload *

Mark only one oval.

- Performance
- Temporal demand

30. Which of the two item is more important contributor to your workload *

Mark only one oval.

- Physical demand
- Performance

31. Which of the two item is more important contributor to your workload *

Mark only one oval.

- Temporal demand
- Mental demand

32. Which of the two item is more important contributor to your workload *

Mark only one oval.

- Mental demand
- Effort

33. Which of the two item is more important contributor to your workload *

Mark only one oval.

- Mental demand
- Physical demand

34. Which of the two item is more important contributor to your workload *

Mark only one oval.

- ( ) Effort
- ( ) Phsical demand

35. Which of the two item is more important contributor to your workload *

Mark only one oval.

- ( ) Frustation
- ( ) Mental demand

Rating scale

Evaluate your workload by rating on the scale of 10. 1 represents least workload, 20 represents highest workload.

36. How mental demanding is your work? *

____

37. How physical demanding is your work? *

____

38. How hurried or rush is your work? *

____

39. How successful are you in accomplishing your work? *

____

40. How hard do you have to work to accomplish your work? *

____

41. How insecure, stressed, discouraging, irritating, annoying is your work? *

____

Practice of respectful maternity care

Protection from physical harm and damage

42. You have never used physical force or abrasive behavior with women (before 2019 January/February *

Mark only one oval.

- Always
- Sometimes
- Never

43. You have never used physical force or abrasive behavior with women. (after 2019 January/February) *

Mark only one oval.

- Always
- Sometimes
- Never

44. You have never physically restrained the women (before 2019 January/February) *

Mark only one oval.

- Always
- Sometimes
- Never

45. You have never physically restrained the women. (after COVID-19) *

Mark only one oval.

- Always
- Sometimes
- Never

46. You have touched or demonstrated touching in a respectful and culturally appropriate way (before 2019 January/February) *

Mark only one oval.

- Always
- Sometimes
- Never

47. You have touched or demonstrated touching in a respectful and culturally appropriate way. (after COVID-19) *

Mark only one oval.

- Always
- Sometimes
- Never

48. You have never separated a woman from her baby, except when it was medically required (before 2019 January/February) *

Mark only one oval.

- Always
- Sometimes
- Never

49. You have never separated a woman from her baby, except when it was medically required (after 2019 January/February) *

Mark only one oval.

- Always
- Sometimes
- Never

50. You always provide or encourage for food or fluid to women in labour, except when it restricted in certain medical condition (before COVID-19 pandemic) *

Mark only one oval.

- Always
- Sometimes
- Never

51. You always provide or encourage for food or fluid to women in labour, except when it restricted in certain medical condition (after 2019 January/February) *

Mark only one oval.

- Always
- Sometimes
- Never

52. You have provided pain-relief and comfort to the women (before 2019 January/February) *

Mark only one oval.

- Always
- Sometimes
- Never

53. You have provided pain-relief and comfort to the women. (after 2019 January/February) *

Mark only one oval.

- Always
- Sometimes
- Never

Consented care

54. Even though you are busy and have workload, you introduce yourself to the women and her companion. (before 2019 January/February) *

Mark only one oval.

- Always
- Sometimes
- Never

55. Even though you are busy and have workload, you introduce yourself to the women and her companion. (after 2019 January/February) *

Mark only one oval.

- Always
- Sometimes
- Never

56. You encourage companion to remain with women whenever possible during the labour (before 2019 January/February) *

Mark only one oval.

- Always
- Sometimes
- Never

57. You encourage companion to remain with women whenever possible during the labour (after 2019 January/February) *

Mark only one oval.

- Always
- Sometimes
- Never

58. Even though you are busy and have workload, you encourage women and her companion to ask questions (before 2019 January/February) *

Mark only one oval.

- Always
- Sometimes
- Never

59. Even though you are busy and have workload, you encourage women and her companion to ask questions. (after 2019 January/February) *

Mark only one oval.

- Always
- Sometimes
- Never

60. If you are asked questions, Even though you are busy and have workload, you respond to question with promptness, politeness and truthfulness (before 2019 January/February) *

Mark only one oval.

- Always
- Sometimes
- Never

61. If you are asked questions, Even though you are busy and have workload, you respond to question with promptness, politeness and truthfulness. (after 2019 January/February) *

Mark only one oval.

- Always
- Sometimes
- Never

62. Even though you are busy and have workload, you always explain to the women what is being done and what to expect throughout the labor and birth. (before 2019 January/February) *

Mark only one oval.

- Always
- Sometimes
- Never

63. Even though you are busy and have workload, you always explain to the women what is being done and what to expect throughout the labor and birth. (after 2019 January/February) *

Mark only one oval.

- Always
- Sometimes
- Never

64. Even though you are busy and have workload, you give periodic updates on status and progress of labor. (before 2019 January/February) *

Mark only one oval.

- Always
- Sometimes
- Never

65. Even though you are busy and have workload, you give periodic updates on status and progress of labor. (after 2019 January/February) *

Mark only one oval.

- Always
- Sometimes
- Never

66. You allow women to move during the labor. (before 2019 January/February) *

Mark only one oval.

- Always
- Sometimes
- Never

67. You allow women to move during the labor. (after 2019 January/February) *

Mark only one oval.

- Always
- Sometimes
- Never

68. You allow women to choose the birthing position. Is there a trend to do delivery in positions other than lithotomy? (before 2019 January/February) *

Mark only one oval.

- Always
- Sometimes
- Never

69. You allow women to choose the birthing position. Is there a trend to do delivery in positions other than lithotomy? (after 2019 January/February) *

Mark only one oval.

- Always
- Sometimes
- Never

70. You obtain consent or permission before doing any procedure on women. (before 2019 January/February) *

Mark only one oval.

- Always
- Sometimes
- Never

71. You obtain consent or permission before doing any procedure on women. (after 2019 January/February) *

Mark only one oval.

- Always
- Sometimes
- Never

Confidential care

72. You store the patient files in locked cabinets with limited access. (before 2019 January/February) *

Mark only one oval.

- Always
- Sometimes
- Never

73. You store the patient files in locked cabinets with limited access. (after 2019 January/February) *

Mark only one oval.

- Always
- Sometimes
- Never

74. You use curtain or visual barrier to protect women during examination, births or any procedure. (before 2019 January/February) *

Mark only one oval.

- Always
- Sometimes
- Never

75. You use curtain or visual barrier to protect women during examination, births or any procedure. (after 2019 January/February) *

Mark only one oval.

- Always
- Sometimes
- Never

76. You use drapes or covering appropriately to protect women's privacy. (before 2019 January/February) *

Mark only one oval.

- Always
- Sometimes
- Never

77. You use drapes or covering appropriately to protect women's privacy. (after 2019 January/February). *

Mark only one oval.

- Always
- Sometimes
- Never

Dignified care

78. You speak politely to women and her companion. (before 2019 January/February) *

Mark only one oval.

- Always
- Sometimes
- Never

79. You speak politely to women and her companion. (after 2019 January/February). *

Mark only one oval.

- Always
- Sometimes
- Never

80. You allow women and her companion to observe cultural practice as much as possible. (before 2019 January/February) *

Mark only one oval.

- Always
- Sometimes
- Never

81. You allow women and her companion to observe cultural practice as much as possible. (after 2019 January/February) *

Mark only one oval.

- Always
- Sometimes
- Never

82. You have never insulted, intimidated, threatened or coerced women and her companion. (before 2019 January/February) *

Mark only one oval.

- Always
- Sometimes
- Never

83. You have never insulted, intimidated, threatened or coerced women and her companion. (after 2019 January/February) *

Mark only one oval.

- Always
- Sometimes
- Never

Non-discriminative care

84. You speak to women at a language level that she understands. (before 2019 January/February) *

Mark only one oval.

- Always
- Sometimes
- Never

85. You speak to women at a language level that she understands. (after 2019 January/February) *

Mark only one oval.

- Always
- Sometimes
- Never

86. You never show disrespect to women based on any specific attribute. (before 2019 January/February) *

Mark only one oval.

- Always
- Sometimes
- Never

87. You never show disrespect to women based on any specific attribute. (after 2019 January/February) *

Mark only one oval.

- Always
- Sometimes
- Never

Non-abandonment care

88. You encourage women and her companion to call whenever required. (before 2019 January/February *

Mark only one oval.

- Always
- Sometimes
- Never

89. You encourage women and her companion to call whenever required. (after 2019 January/February) *

Mark only one oval.

- Always
- Sometimes
- Never

90. You come quickly when women call. (before 2019 January/February) *

Mark only one oval.

- Always
- Sometimes
- Never

91. You come quickly when women call. (after 2019 January/February) *

Mark only one oval.

- Always
- Sometimes
- Never

92. You have never left women alone or unattended. (before 2019 January/February) *

Mark only one oval.

- Always
- Sometimes
- Never

93. You have never left women alone or unattended. (after 2019 January/February) *

Mark only one oval.

- Always
- Sometimes
- Never

This content is neither created nor endorsed by Google.

Google Forms

नेपालमा सवास्थाकर्मिमा कामको भार र आदर प्रसूति हेरचार अभ्यास बिचको सम्बन्ध

प्रश्नावली

* Required

सर्वे प्रश्नावली , नेपालीमा

1. अन्तर्वार्ताकर्ताको पहिचान नम्बर (टेलिफोन नम्बर) अथवा परिचय नम्बर *

____

2. कार्यरत जिल्लाको नाम *

____

3. कार्यरत स्वस्थासंस्थाको नाम *

____

4. कार्यरत नगरको तह *

Mark only one oval.

( ) उप- महानगरपालिका

( ) नगरपालिका

( ) गाउपालिका

5. कार्यरत स्वस्थासंस्थाको किसिम *

Mark only one oval.

- ⬭ प्रदेश अस्पताल
- ⬭ जिल्ला अस्पताल
- ⬭ नगर अस्पताल
- ⬭ प्राथमिक स्वास्थ केन्द्र
- ⬭ हेल्थ पोष्ट
- ⬭ नगर स्वास्थ केन्द्र
- ⬭ बिर्थिङ्ग सेन्टर
- ⬭ अन्य

जनसांख्यिकी, पेशा तथा कार्य सम्बन्धित जानकारी

6. तपाईको उमेर (पुरा गरेको वर्ष) *

____

7. तपाईले पुरा गरेको उच्च शिक्षा *

Mark only one oval.

- ⬭ मातृ तथा बच्चा सम्बन्धि तालिम
- ⬭ अ . न. मि
- ⬭ स्टाफ नर्स
- ⬭ स्नातक तह नर्स
- ⬭ स्नातक उत्तर तह नर्स
- ⬭ अन्य पारामेडिक्स

8. तपाईको अहिलेको पेशा *

Mark only one oval.

- मातृ शिशु कार्यकर्ता
- अ . न. मि
- स्टाफ नर्स
- नर्सिङ्ग अफिसर
- नर्सिङ्ग प्रशासक
- अन्य

9. तपाईको कार्य अबधि , वर्षमा *

10. अहिलेको कार्य स्थलमा काम गरेको कति वर्ष भयो *

11. के तपाइले एस. बि. ए (SBA) तालिम लिनुभएको छ ? *

Mark only one oval.

- छ
- छैन

12. के तपाइले कहिले पनि आदर प्रसुति सेवा बारे सुन्नु भएको थियो अथवा पढ्नु भएको थियो ? *

Mark only one oval.

- पढेको / सुनेको छु
- पढेको / सुनेको छैन
- याद भएन

13. सुन्नु भएको थियो अथवा पढ्नु भएको थियो भने , कहाँ पढ्नु/सुन्नु भएको थियो?

Mark only one oval.

- ( ) क्याम्पस / बिधालय /university मा पढ्दा
- ( ) तालिममा
- ( ) अन्य
- ( ) Other: ____________________

14. के तपाई कोभिड संक्रमित हुनुभएको थियो ? येदि भन्न चाहनुहुन्छ भने ? *

Mark only one oval.

- ( ) थिए
- ( ) थिएन
- ( ) भन्न चाहन्न

स्वास्थ्यकर्मिको कार्य सम्बन्धि विवरण

15. अनुमानित प्रसूतीको गरिएको संख्या (पछिल्लो १ हप्तामा). *

____

16. अनुमानित प्रसूतीको गरिएको संख्या (पछिल्लो १ महिनामा) *

____

17. अनुमानित अनुमानित प्रसूतीको गरिएको संख्या (पछिल्लो ६ महिनामा) *

____

18. अनुमानित एक्सटरा *extra* डियुटि गरिएको संख्या (पछिल्लो १ हप्तामा) *

____

19. अनुमानित एक्सटरा *extra* डियुटि गरिएको संख्या (पछिल्लो १ महिनामा) *

20. एक्सटरा *extra* डियुटि गरिएको संख्या (पछिल्लो ६ महिनामा) *

कार्य भारको मापन (कथित कार्यभारको मूल्याङ्कन)

21. तलका दुई मध्ये कुन विशेषताहरूले तपाइँको काम अथवा कामको भारलाई बेसी वर्णन गर्दछ *

Mark only one oval.

- मेहेनत बेसी छ
- काम राम्रो संग सकाउनु पर्छ भन्ने दबाब बेसी छ

22. तलका दुई मध्ये कुन विशेषताहरूले तपाइँको काम अथवा कामको भारलाई बेसी वर्णन गर्दछ *

Mark only one oval.

- काममा निकै हतार हुन्छ / काममा निकै समय को दबाब हुन्छ
- तनाब बेसी छ

23. तलका दुई मध्ये कुन विशेषताहरूले तपाइँको काम अथवा कामको भारलाई बेसी वर्णन गर्दछ *

Mark only one oval.

- तनाब बेसी छ
- मेहेनत बेसी छ

24. तलका दुई मध्ये कुन विशेषताहरूले तपाइँको काम अथवा कामको भारलाई बेसी वर्णन गर्दछ *

Mark only one oval.

- काममा निकै हतार हुन्छ / काममा निकै समय को दबाब हुन्छ
- मेहेनत बेसी छ

25. तलका दुई मध्ये कुन विशेषताहरूले तपाइँको काम अथवा कामको भारलाई बेसी वर्णन गर्दछ *

Mark only one oval.

- शारीरिक काम बेसी छ
- तनाब बेसी छ

26. तलका दुई मध्ये कुन विशेषताहरूले तपाइँको काम अथवा कामको भारलाई बेसी वर्णन गर्दछ *

Mark only one oval.

- काम राम्रो संग सकाउनु पर्चा भन्ने दबाब बेसी छ
- मानसिक काम बेसी छ

27. तलका दुई मध्ये कुन विशेषताहरूले तपाइँको काम अथवा कामको भारलाई बेसी वर्णन गर्दछ *

Mark only one oval.

- काम राम्रो संग सकाउनु पर्चा भन्ने दबाब बेसी छ
- तनाब बेसी छ

28. तलका दुई मध्ये कुन विशेषताहरूले तपाइँको काम अथवा कामको भारलाई बेसी वर्णन गर्दछ *

Mark only one oval.

- काममा निकै हतार हुन्छ / काममा निकै समय को दबाब हुन्छ
- शारीरिक कम बेसी छ

29. तलका दुई मध्ये कुन विशेषताहरूले तपाइँको काम अथवा कामको भारलाई बेसी वर्णन गर्दछ *

Mark only one oval.

- काममा निकै हतार हुन्छ / काममा निकै समय को दबाब हुन्छ
- काम राम्रो संग सकाउनु पर्चा भन्ने दबाब बेसी छ

30. तलका दुई मध्ये कुन विशेषताहरूले तपाइँको काम अथवा कामको भारलाई बेसी वर्णन गर्दछ *

Mark only one oval.

- काम राम्रो संग सकाउनु पर्चा भन्ने दबाब बेसी छ
- शारीरिक काम बेसी छ

31. तलका दुई मध्ये कुन विशेषताहरूले तपाइँको काम अथवा कामको भारलाई बेसी वर्णन गर्दछ *

Mark only one oval.

- काममा निकै हतार हुन्छ / काममा निकै समय को दबाब हुन्छ
- काममा मानसिक काम बेसी छ

32. तलका दुई मध्ये कुन विशेषताहरूले तपाइँको काम अथवा कामको भारलाई बेसी वर्णन गर्दछ *

Mark only one oval.

- काममा मानसिक काम बेसी छ
- मेहेनत बेसी छ

33. तलका दुई मध्ये कुन विशेषताहरूले तपाइँको काम अथवा कामको भारलाई बेसी वर्णन गर्दछ *

Mark only one oval.

- शारीरिक काम बेसी छ
- मेहेनत बेसी छ

34. तलका दुई मध्ये कुन विशेषताहरूले तपाइँको काम अथवा कामको भारलाई बेसी वर्णन गर्दछ *

Mark only one oval.

- तनाब बेसी छ
- मानसिक काम बेसी छ

35. तलका दुई मध्ये कुन विशेषताहरूले तपाइँको काम अथवा कामको भारलाई बेसी वर्णन गर्दछ *

Mark only one oval.

- [ ] मानसिक काम बेसी छ
- [ ] शारीरिक काम बेसी छ

कार्यभार रेटिंग स्केल

36. तपाईको कामको लागि कति मानसिक र बौद्धिक गतिविधि आवश्यक छ ? १ देखि २० नम्बर भित्र मुल्यांकन गर्नुहोस। एक भनेको सबै भन्दा कम , २० भनेको सबै भन्दा बेसी , १० भनेको मध्ये। *

____

37. तपाईको काममा कति समय दबाब/हतार छ? १ देखि २० नम्बर भित्र मुल्यांकन गर्नुहोस। एक भनेको सबै भन्दा कम , २० भनेको सबै भन्दा बेसी , १० भनेको मध्ये। *

____

38. तपाई आफ्नो कार्य पूरा गर्नमा कत्तिको सफल भएको जस्तो लाग्छ? १ देखि २० नम्बर भित्र मुल्यांकन गर्नुहोस। एक भनेको सबै भन्दा कम , २० भनेको सबै भन्दा बेसी , १० भनेको मध्ये। *

____

39. तपाईको काम पूरा गर्न कति प्रयास /मेहेनत गर्नुपर्दछ? १ देखि २० नम्बर भित्र मुल्यांकन गर्नुहोस। एक भनेको सबै भन्दा कम , २० भनेको सबै भन्दा बेसी , १० भनेको मध्ये। *

____

40. तपाईको कामको लागि कति शारीरिक गतिविधि आवश्यक छ ?१ देखि २० नम्बर भित्र मुल्यांकन गर्नुहोस। एक भनेको सबै भन्दा कम , २० भनेको सबै भन्दा बेसी , १० भनेको मध्ये। *

____

41. तपाईको काम कत्तिको तनावपूर्ण, झर्कोलाग्दो , निराश पार्ने छ ?१ देखि २० नम्बर भित्र मुल्यांकन गर्नुहोस। एक भनेको सबै भन्दा कम , २० भनेको सबै भन्दा बेसी , १० भनेको मध्ये। *

____

आदर प्रसूती हेरचार अभ्यास *(Respectful maternity care practice)*

शारीरिक क्षति र उपचारबाट सुरक्षा (Protection from mental and physical harm)

42. तपाईले कहिल्यै महिलासँग रुखो स्वोरमा बोल्नुहुन्न अथवा शारीरिक बल वा रुखो व्यवहार गर्नुहुन्न (कोभिड अगाडी) *

Mark only one oval.

- सधैं राम्रो संग बोल्छु ,
- कहिलेकाँही राम्रो संग बोल्छु ,
- कहिले पनि राम्रो संग बोल्दिन ,

43. तपाईले कहिल्यै महिलासँग रुखो स्वोरमा बोल्नुहुन्न अथवा शारीरिक बल वा रुखो व्यवहार गर्नुहुन्न (कोभिड पछाडी) *

Mark only one oval.

- सधैं सधैं राम्रो संग बोल्छु
- कहिलेकाँही राम्रो संग बोल्छु
- कहिले पनि राम्रो संग बोल्दिन

44. तपाईंले महिलालाई कहिले पनि शारीरिक बन्धनमा राख्नुहुन्न। (कोभिड अगाडी) *

Mark only one oval.

- कहिले पनि राख्दिन
- कहिलेकाँही राख्छु
- सधै राख्छु

45. तपाईंले महिलालाई कहिले पनि शारीरिक बन्धनमा राख्नुहुन्न। (कोभिड पछाडी) *

Mark only one oval.

- कहिले पनि राख्दिन
- कहिलेकाँही राख्छु
- सधै राख्छु

46. तपाईले मर्यादित रुपमा मात्र महिलालाई छुनु हुन्छ। (कोभिड अगाडी) *

Mark only one oval.

- सधैं
- कहिलेकाँही
- कहिले पनि होइन

47. तपाईले मर्यादित रुपमा मात्र महिलालाई छुनु हुन्छ।(कोभिद पछाडी) *

Mark only one oval.

- सधैं
- कहिलेकाँही
- कहिले पनि होइन

48. तपाईंले एक महिलालाई उनको बच्चाबाट कहिले पनि अलग गर्नुभएको छैन, मेडिकल आवश्यकता बाहेक (कोभिद अगाडी) *

Mark only one oval.

- सधैं संगै राख्छु
- कहिलेकाँही मात्र राख्छु
- कहिले पनि संगै राख्दिन

49. तपाईंले एक महिलालाई उनको बच्चाबाट कहिले पनि अलग गर्नुभएको छैन, मेडिकल आवश्यकता बाहेक (कोभिद पछाडी) *

Mark only one oval.

- सधैं संगै राख्छु
- कहिलेकाँही मात्र राख्छु
- कहिले पनि संगै राख्दिन

50. तपाईले प्रसव पिडामा भएकी महिलालाई सधै खाना र तरल पदार्थ दिनुहुन्छ अथवा खानलाई प्रोत्साहन गर्नुहुन्छ ? मेडिकल आवस्यकता बाहेक (कोभिद अगाडी) *

Mark only one oval.

- सधैं
- कहिलेकाँही
- कहिले पनि होइन

51. तपाईले प्रसव पिडामा भएकी महिलालाई सधै खाना र तरल पदार्थ दिनुहुन्छ अथवा खानलाई प्रोत्साहन गर्नुहुन्छ ? मेडिकल आवस्यकता बाहेक (कोभिद पछाडी) *

Mark only one oval.

- सधैं
- कहिलेकाँही
- कहिले पनि होइन

52. तपाईंले प्रसव पिडामा भएकी महिलालाई पीडा-राहत र आराम प्रदान गर्नुहुन्छ । (कोभिद अगाडी) *

Mark only one oval.

- सधैं
- कहिलेकाँही
- कहिले पनि होइन

53. तपाईंले प्रसव पिडामा भएकी महिलालाई पीडा-राहत र आराम प्रदान गर्नुहुन्छ । (कोभिद पछाडी) *

Mark only one oval.

- सधैं
- कहिलेकाँही
- कहिले पनि होइन

सहमतिपूर्ण हेरचाह (consented care)

54. जस्तो बेस्तता अथवा कामको बोझ भए पनि , तपाईंले महिला र उनको साथीलाई आफ्नो परिचय दिनुहुन्छ ? (कोभिद अगाडी) *

Mark only one oval.

- सधैं
- कहिलेकाँही
- कहिले पनि होइन

55. जस्तो बेस्तता अथवा कामको बोझ भए पनि , तपाईंले महिला र उनको साथीलाई आफ्नो परिचय दिनुहुन्छ ? (कोभिद पछाडी) *

Mark only one oval.

- सधैं
- कहिलेकाँही
- कहिले पनि होइन

56. तपाईं सम्भव भएसम्म महिलाको साथीहरूलाई अथवा परिवारलाई प्रसव अवस्था भरि महिलाको साथ रहन प्रोत्साहित गर्नुहुन्छ ? (कोभिद अगाडी) *

Mark only one oval.

- सधैं
- कहिलेकाँही
- कहिले पनि होइन

57. तपाईं सम्भव भएसम्म महिलाको साथीहरूलाई अथवा परिवारलाई प्रसव अवस्था भरि महिलाको साथ रहन प्रोत्साहित गर्नुहुन्छ ? (कोभिद पछाडी) *

Mark only one oval.

- सधैं
- कहिलेकाँही
- कहिले पनि होइन

58. जस्तो बेस्तता अथवा कामको बोझ भएपनि ,तपाईंले महिला र उनको साथीलाई प्रश्न सोध्न प्रोत्साहित गर्नुहुन्छ। (कोभिड अगाडी) *

Mark only one oval.

- सधैं
- कहिलेकाँही
- कहिले पनि होइन

59. जस्तो बेस्तता अथवा कामको बोझ भएपनि ,तपाईंले महिला र उनको साथीलाई प्रश्न सोध्न प्रोत्साहित गर्नुहुन्छ। (कोभिड पछाडी) *

Mark only one oval.

- सधैं
- कहिलेकाँही
- कहिले पनि होइन

60. येदि महिला र उनको साथीले प्रश्न सोध्नु भयो भने , जस्तो बेस्तता अथवा कामको बोझ भएपनि ,तपाईंले तुरुन्त, शिष्टता र सत्यता संग प्रश्नको जवाफ दिनुहुन्छ। (कोभिड अगाडी) *

Mark only one oval.

- सधैं
- कहिलेकाँही
- कहिले पनि होइन

61. येदि महिला र उनको साथीले प्रश्न सोध्नु भयो भने , जस्तो बेस्तता अथवा कामको बोझ भएपनि ,तपाईंले तुरुन्त, शिष्टता र सत्यता संग प्रश्नको जवाफ दिनुहुन्छ। (कोभिड पछाडी) *

Mark only one oval.

- सधैं
- कहिलेकाँही
- कहिले पनि होइन

62. जस्तो बेस्तता अथवा कामको बोझ भएपनि ,तपाईंले जहिले पनि महिलालाई के हुँदैछ र प्रसवभरि के आशा गर्ने भनेर बुझाउनुहुन्छ। (कोभिड अगाडी) *

Mark only one oval.

- सधैं
- कहिलेकाँही
- कहिले पनि होइन

63. जस्तो बेस्तता अथवा कामको बोझ भएपनि ,तपाईंले जहिले पनि महिलालाई के हुँदैछ र प्रसवभरि के आशा गर्ने भनेर बुझाउनुहुन्छ। (कोभिड पछाडी) *

Mark only one oval.

- सधैं
- कहिलेकाँही
- कहिले पनि होइन

64. जस्तो बेस्तता अथवा कामको बोझ भएपनि ,तपाईंले प्रसवको स्थिति र प्रगतिमा महिलालाई अपडेट दिनुहुन्छ। (कोभिड अगाडी) *

Mark only one oval.

- सधैं
- कहिलेकाँही
- कहिले पनि होइन

65. जस्तो बेस्तता अथवा कामको बोझ भएपनि ,तपाईंले प्रसवको स्थिति र प्रगतिमा महिलालाई अपडेट दिनुहुन्छ (कोभिड पछाडी) *

Mark only one oval.

- सधैं
- कहिलेकाँही
- कहिले पनि होइन

66. तपाइँ महिलालाई प्रसवको बखत चल्न वा हल्लिन अनुमति दिनुहुन्छ। (कोभिड अगाडी) *

Mark only one oval.

- सधैं
- कहिलेकाँही
- कहिले पनि होइन

67. तपाइँ महिलालाई प्रसवको बखत चल्न वा हल्लिन अनुमति दिनुहुन्छ। (कोभिड अगाडी) *

Mark only one oval.

- सधैं
- कहिलेकाँही
- कहिले पनि होइन

68. तपाइले महिलालाई उनले चाहेको बर्थ पोजिशनमा देलिवेरी गर्न दिनुहुन्छ ? अथवा के लिठोतोमी बाहेक अरु पोजिशन मा देलिवेरी गराउने चलन छ ? (कोभिद अगाडी) *

Mark only one oval.

- सधैं
- कहिलेकाँही
- कहिले पनि होइन

69. तपाइले महिलालाई उनले चाहेको बर्थ पोजिशन मा देलिवेरी गर्न दिनुहुन्छ ? अथवा के लिठोतोमी बाहेक अरु पोजिशन मा देलिवेरी गराउने चलन छ ? (कोविड़ पछाडी) *

Mark only one oval.

- सधैं
- कहिलेकाँही
- कहिले पनि होइन

70. महिलामा कुनै प्रक्रिया गर्नु अघि तपाईले महिलासँग सहमति वा अनुमति प्राप्त गर्नुहुन्छ। (कोभिद अगाडी) *

Mark only one oval.

- सधैं
- कहिलेकाँही
- कहिले पनि होइन

71. महिलामा कुनै प्रक्रिया गर्नु अघि तपाईले महिलासँग सहमति वा अनुमति प्राप्त गर्नुहुन्छ।(कोभिद पछाडी) *

Mark only one oval.

- सधैं
- कहिलेकाँही
- कहिले पनि होइन

गोपनीय हेरचाह (Confidentiality)

72. तपाईं सीमित पहुँचको हुने ठाउँ वा लक क्याबिनेटहरुमा मात्र बिरामी फाइलहरू भण्डारण गर्नुहुन्छ।(कोभिद अगाडी) *

Mark only one oval.

- ( ) सधैं
- ( ) कहिलेकाँही
- ( ) कहिले पनि होइन

73. तपाईं सीमित पहुँचको हुने ठाउँ वा लक क्याबिनेटहरुमा मात्र बिरामी फाइलहरू भण्डारण गर्नुहुन्छ।(कोभिद पछाडी) *

Mark only one oval.

- ( ) सधैं
- ( ) कहिलेकाँही
- ( ) कहिले पनि होइन

74. तपाईं महिलालाई परिक्षण गर्ने बखत पर्दा वा गोपनियता राख्नको लागि कुनै अवरोधको प्रयोग गर्नुहुन्छ? (कोभिद अगाडी) *

Mark only one oval.

- ( ) सधैं
- ( ) कहिलेकाँही
- ( ) कहिले पनि होइन

75. तपाईं महिलालाई परिक्षण गर्ने बखत पर्दा वा गोपनियता राख्नको लागि कुनै अवरोधको प्रयोग गर्नुहुन्छ? (कोभिद पछाडी) *

Mark only one oval.

- ( ) सधैं
- ( ) कहिलेकाँही
- ( ) कहिले पनि होइन

76. तपाईं महिलाको गोपनीयता सुरक्षित गर्नको लागि *procedure* गर्ने बखत ड्रेप्स वा कभरिंग प्रयोग गर्नुहुन्छ।(कोभिद अगाडी) *

Mark only one oval.

- सधैं
- कहिलेकाँही
- कहिले पनि होइन

77. तपाईं महिलाको गोपनीयता सुरक्षित गर्नको लागि *procedure* गर्ने बखत ड्रेप्स वा कभरिंग प्रयोग गर्नुहुन्छ।(कोभिद पछाडी डी) *

Mark only one oval.

- सधैं
- कहिलेकाँही
- कहिले पनि होइन

सम्मानित हेरचाह (dignified care)

78. तपाईं महिला र उनको साथीसंग सधैँ नम्रतापूर्वक बोल्नुहुन्छ।(कोभिद अगाडी) *

Mark only one oval.

- सधैं
- कहिलेकाँही
- कहिले पनि होइन

79. तपाईं महिला र उनको साथीसंग सधै नम्रतापूर्वक बोल्नुहुन्छ।(कोभिद पछाडी डी) *

Mark only one oval.

- सधैं
- कहिलेकाँही
- कहिले पनि होइन

80. तपाईं महिला र उनको साथीलाई मिलेसम्म वाहाहरुको धार्मिक तथा सांस्कृतिक अभ्यास गर्नको लागि अनुमति दिनुहुन्छ। (कोभिद अगाडी) *

Mark only one oval.

- सधैं
- कहिलेकाँही
- कहिले पनि होइन

81. तपाईं महिला र उनको साथीलाई मिलेसम्म वाहाहरुको धार्मिक तथा सांस्कृतिक अभ्यास गर्नको लागि अनुमति दिनुहुन्छ।(कोभिद पछाडी) *

Mark only one oval.

- सधैं
- कहिलेकाँही
- कहिले पनि होइन

82. तपाईं महिला र उनको साथीलाई कहिले पनि अपमान, डर, धम्की वा जबरजस्ती गर्नुहुन्न। (कोभिद अगाडी) *

Mark only one oval.

- सधैं गर्दिन
- कहिलेकाँही गर्छु
- सधै गर्छु

83. तपाईं महिला र उनको साथीलाई कहिले पनि अपमान, डर, धम्की वा जबरजस्ती गर्नुहुन्न। (कोविद पछाडी) *

Mark only one oval.

- सधैं गर्दिन
- कहिलेकाँही गर्छु
- सधै गर्छु

भेदभावमुक्त हेरचाह (Non-discriminative care)

84. तपाईं जहिले पनि महिलाले र महिलाको परिवारले बुझ्ने भाषामा बोल्नुहुन्छ। (कोभिद अगाडी) *

Mark only one oval.

- सधैं
- कहिलेकाँही
- कहिले पनि होइन

85. तपाईं जहिले पनि महिलाले र महिलाको परिवारले बुझ्ने भाषामा बोल्नुहुन्छ। (कोभिद पछाडी) *

Mark only one oval.

- सधैं
- कहिलेकाँही
- कहिले पनि होइन

86. तपाईले कुनै पनि आधारमा महिलालाई अनादर अथवा भेदभाव गर्नुहुन्न। (कोभिद अगाडी) *

Mark only one oval.

- सधैं गर्दिन
- कहिलेकाँही गर्छु
- सधै गर्छु

87. तपाईले कुनै पनि आधारमा महिलालाई अनादर अथवा भेदभाव गर्नुहुन्न। ((कोभिद पछाडी) *

Mark only one oval.

- सधैं गर्दिन
- कहिलेकाँही गर्छु
- कहिले पनि गर्दिन

परित्यागमुक्त हेरचाह (Non-abandonment care)

88. तपाईं महिला र उनको साथीलाई आवश्यक पर्दा तपाईहरुलाई बोलाउन अथवा कल गर्न प्रोत्साहित गर्नुहुन्छ। (कोभिद अगाडी) *

Mark only one oval.

- सधैं
- कहिलेकाँही
- कहिले पनि होइन

89. तपाईं महिला र उनको साथीलाई आवश्यक पर्दा तपाईहरुलाई बोलाउन अथवा कल गर्न प्रोत्साहित गर्नुहुन्छ। (कोभिद पछाडी) *

Mark only one oval.

- सधैं
- कहिलेकाँही
- कहिले पनि होइन

90. तपाईं महिला र उनको साथीलाई आवश्यक पर्दा तपाईहरुलाई बोलाउनु भयो भने चाँडै आउनुहुन्छ (कोभिद अगाडी) *

Mark only one oval.

- सधैं
- कहिलेकाँही
- कहिले पनि होइन

91. तपाईं महिला र उनको साथीलाई आवश्यक पर्दा तपाईहरुलाई बोलाउनु भयो भने चाँडै आउनुहुन्छ *
(कोभिद पछाडी)

Mark only one oval.

- सधैं
- कहिलेकाँही
- कहिले पनि होइन

92. तपाइले प्रसवमा भएकी महिलालाई कहिले पनि एक्लै छोड्नु हुन्न। (कोभिद अगाडी)

Mark only one oval.

- सधैं
- कहिलेकाँही
- कहिले पनि होइन

93. तपाइले प्रसवमा भएकी महिलालाई कहिले पनि एक्लै छोड्नु हुन्न। (कोभिद पछाडी)

Mark only one oval.

- सधैं
- कहिलेकाँही
- कहिले पनि होइन

This content is neither created nor endorsed by Google.

Google Forms
